# Supplementary material for: Identification and Characterization of Hdh-FMRF2 Gene in Pacific Abalone and Its Possible Role in Reproduction and Larva Development
Source: Biomolecules. 2023 Jan 5;13(1):109. doi: 10.3390/biom13010109 (PMC9856054; doi:10.3390/biom13010109)
Supplement: Supplementary file 1 [file biomolecules-13-00109-s001.zip › Supplementary Table S1.pdf]

Identification and Characterization of Hdh-FMRF2 gene in Pacific Abalone and Its Possible Role in Reproduction and Larva Development

Supplementary Table S1. List of primers used for cDNA cloning, *in-situ* hybridization, and qRT-PCR analysis of *Hdh-FMRF2* in Pacific abalone.

**Primer Name**	**Nucleotide sequence (5′--- 3′)**	**Purpose**
Oligo dT (OdT)	GGCCACGCGTCGACTAGTACTTTTTTTTTTTTTTTTT	cDNA Synthesis
Oligo dT Adaptor (AP)	GGCCACGCGTCGACTAGTAC	
FMRF2 – Fw	GAGGACAAAGCCTACCTACG	RT-PCR
FMRF2 – Rv	CCTACGAGGACAAAGCCTAC	
Hdh-FMRF2 – 3′RACE	GATTACGCCAAGCTTTGCTAACTATGACGCTCTGAGCGACAGG	RACE PCR
Hdh-FMRF2 – 5′RACE	GATTACGCCAAGCTTGCATCACGCTTACGTCGGTATTGCCAG	
Universal Primer (Short)	CTAATACGACTCACTATAGGGC	
Universal Primer (Long)	CTAATACGACTCACTATAGGGCAAGCAGTGGTATCAACGCAGAGT	
Hdh- FMRF2 (q) – Fw	ACAACAACGGCTGGCAATAC	qRT-PCR
Hdh- FMRF2 (q) – Rv	GCTCAGAGCGTCATAGTTAGC	
Hdh-β-Actin – Fw	CCGTGAAAAGATGACCCAGA	
Hdh-β-Actin – Rv	TACGACCGGAAGCGTACAGA	
Hdh- FMRF2 – Anti-sense	AGCAGAAGGCCAAGTTATGC	*In-situ* Hybridization
Hdh- FMRF2 – Sense	TCGGTAAACGTGGATTTGAAG	
