# Supplementary material for: Identification and Characterization of Hdh-FMRF2 Gene in Pacific Abalone and Its Possible Role in Reproduction and Larva Development
Source: Biomolecules. 2023 Jan 5;13(1):109. doi: 10.3390/biom13010109 (PMC9856054; doi:10.3390/biom13010109)
Supplement: Supplementary file 1 [file biomolecules-13-00109-s001.zip › Supplementary Table S2.pdf]

Identification and Characterization of Hdh-FMRF2 gene in Pacific Abalone and Its Possible Role in Reproduction and Larva Development

Supplementary Table S2. Comparison of peptide predicted between Hdh-FMRF1 and Has-FRMF2 precursors.

Species	Transcript and peptide sequences	No. of peptides encoded	Predicted monoisotopic mass (Da)	Isoelectric point	Attribute	Ref.
Haliotis discus hannai	*Pre-tetrabasic cleavage site:*					This study
	FLRFa	1	580.36	10.55	Basic	
	NFGEPFLRFa	1	1,126.28	6.00	Neutral	
	FDSYEDKAYLRFa	1	1,553.69	4.56	Acidic	
	Ac-SDPGEDPMLKAILLRGAPNNNGWQY	1	2,757.07	4.56	Acidic	
	Post-tetrabasic cleavage site:					
	DAMDETKVKDNDHSRQ	1	1,888.98	4.75	Acidic	
	FMRFa	1	598.31	10.55	Basic	
	NGWLHFa	1	772.86	7.55	Basic	
Haliotis asinina	*Pre-tetrabasic cleavage site:*					Cummins et al., 2011
	FLRFa	1	580.36	10.55	Basic	
	NFGEPFLRFa	1	1,125.28	7.81	Neutral	
	FDSYEDKAYLRFa	1	1,551.74	5.71	Acidic	
	Ac-SDPGEDMLKSILLRGAPSNNGLQ	1	2,575.86	4.37	Acidic	
	Post-tetrabasic cleavage site:					
	DTVDETTVNDNAHSRQ	1	1,800.79	4.06	Acidic	
	FMRFa	1	598.31	10.55	Basic	
	NGWLHFa	1	771.80	7.87	Basic	
