# Supplementary material for: Identification and Characterization of Hdh-FMRF2 Gene in Pacific Abalone and Its Possible Role in Reproduction and Larva Development
Source: Biomolecules. 2023 Jan 5;13(1):109. doi: 10.3390/biom13010109 (PMC9856054; doi:10.3390/biom13010109)
Supplement: Supplementary file 1 [file biomolecules-13-00109-s001.zip › Supplementary Table S3.pdf]

Identification and Characterization of Hdh-FMRF2 gene in Pacific Abalone and Its Possible Role in Reproduction and Larva Development

Table S3: Sequence information of FaRPs used for motif analysis, multiple sequence alignment and phylogenetic analysis.

Common Name	Scientific Name	Gene	GenBank Accession No.	
			Nucleotide	Protein
Peltospirid snail	*Gigantopelta aegis*	FMRFamide	XM_041510333	XP_041366267
Pacific abalone	*Haliotis discus hannai*	FMRFamide 1	MF066907	AVW85483
		FMRFamide 2	MZ224009	QXP00688
Tropical abalone	*Haliotis asinina*	FMRFamide 1	EU684321	ACD65487
		FMRFamide 2	EU684322	ACD65488
Pacific oyster	*Crassostrea gigas*	FMRFamide	-	EKC24087
Yesso scallop	*Mizuhopecten yessoensis*	FMRFamide	XM_021523180	XP_021378855
East Asian common octopus	*Octopus sinensis*	FMRFamide	XM_029788266	XP_029644126
California two-spot octopus	*Octopus bimaculoides*	FMRFamide 1	XM_014920442	XP_014775928
		FMRFamide 2	XM_014920445	XP_014775931
Southern Pygmy Squid	*Xipholeptos notoides*	FMRFamide	FJ896403	ACP39631
Longfin inshore squid	*Doryteuthis pealeii*	FMRFamide	FJ205479	ACI22791
Opalescent inshore squid	*Doryteuthis opalescens*	FMRFamide	AF303160	AAG22544
Pharaoh cuttlefish	*Sepia pharaonis*	FMRFamide	KX000397	AQM50872
		LFRFamide	MG869822	QBG58624
Japanese spineless cuttlefish	*Sepiella japonica*	FMRFamide 2	KJ933411	AJT49288
		LFRFamide	KP260902	AKE48167
European common cuttlefish	*Sepia officinalis*	FMRFamide	CAA72116	CAA72116
California sea hare	*Aplysia californica*	FMRFamide	M14958	AAA27755
		LFRFamide	NM_001204500	NP_001191429
Common Garden Snail	*Helix aspersa*	FMRFamide	-	2005361A
Lemon snail	*Cepaea nemoralis*	FMRFamide	U02488	AAA03426
Great Pond Snail	*Lymnaea stagnalis*	FMRFamide	M37629	AAA63280
		LFRFamide	AY773478	AAV41057
Marsh snail	*Biomphalaria glabrata*	FMRFamide 1	QQO98580	QQO98580
		FMRFamide 2	MW825357	QWQ58173
		FMRFamide 3	MW387020	QQO98581
Alexandrina snail	*Biomphalaria alexandrina*	FMRFamide	MK144535	QCX41799
Mediterranean fruit fly	*Ceratitis capitata*	FMRFamide 2	XM_004526012	XP_004526069
Stable fly	*Stomoxys calcitrans*	FMRFamide 1	XM_013242316	XP_013097770
		FMRFamide 2	XM_013242317	XP_013097771
House fly	*Musca domestica*	FMRFamide	AB214648	BAF73475
Fruit fly	*Drosophila mojavensis*	FMRFamide	XM_002004728	XP_002004764
Fruit fly	*Drosophila* sp.	FMRFamide	AH001003	AAA28538
Common fruit fly	*Drosophila melanogaster*	FMRFamide	-	AAF58874
Three-band garden slug	*Ambigolimax valentianus*	LFRFamide 1	LC375313	BBD49867
		LFRFamide 2	LC375314	BBD49868
Grey field slug	*Deroceras reticulatum*	LFRFamide 1	KY659296	ARS01397
		LFRFamide 2	KY659297	ARS01398
Triton's Trumpet	*Charonia tritonis*	LFRFamide 1	KY287993	AQS80524
		LFRFamide 2	KY287994	AQS80525
Human	*Homo sapiens*	FaRP FF	NM_003717	NP_003708
		NP VF	AF330057	AAK94201
Feral cattle	*Bos taurus*	FaRP FF	NM_174123	NP_776548
		NP VF	NM_174168	NP_776593
House mouse	*Mus musculus*	FaRP FF	NM_018787	NP_061257
		NP VF	AF330058	AAK94202
Norway rat	*Rattus norvegicus*	FaRP FF	NM_022586	NP_072108
		NP VF	AF330059	AAK94203
Sheep	*Ovis aries*	NP VF	NM_001127268	NP_001120740
Zebrafish	*Danio rerio*	FaRP1	EF547661	ABR68853
