# Supplementary figures and images for: Identification and Characterization of Hdh-FMRF2 Gene in Pacific Abalone and Its Possible Role in Reproduction and Larva Development
Source: Biomolecules. 2023 Jan 5;13(1):109. doi: 10.3390/biom13010109 (PMC9856054; doi:10.3390/biom13010109)

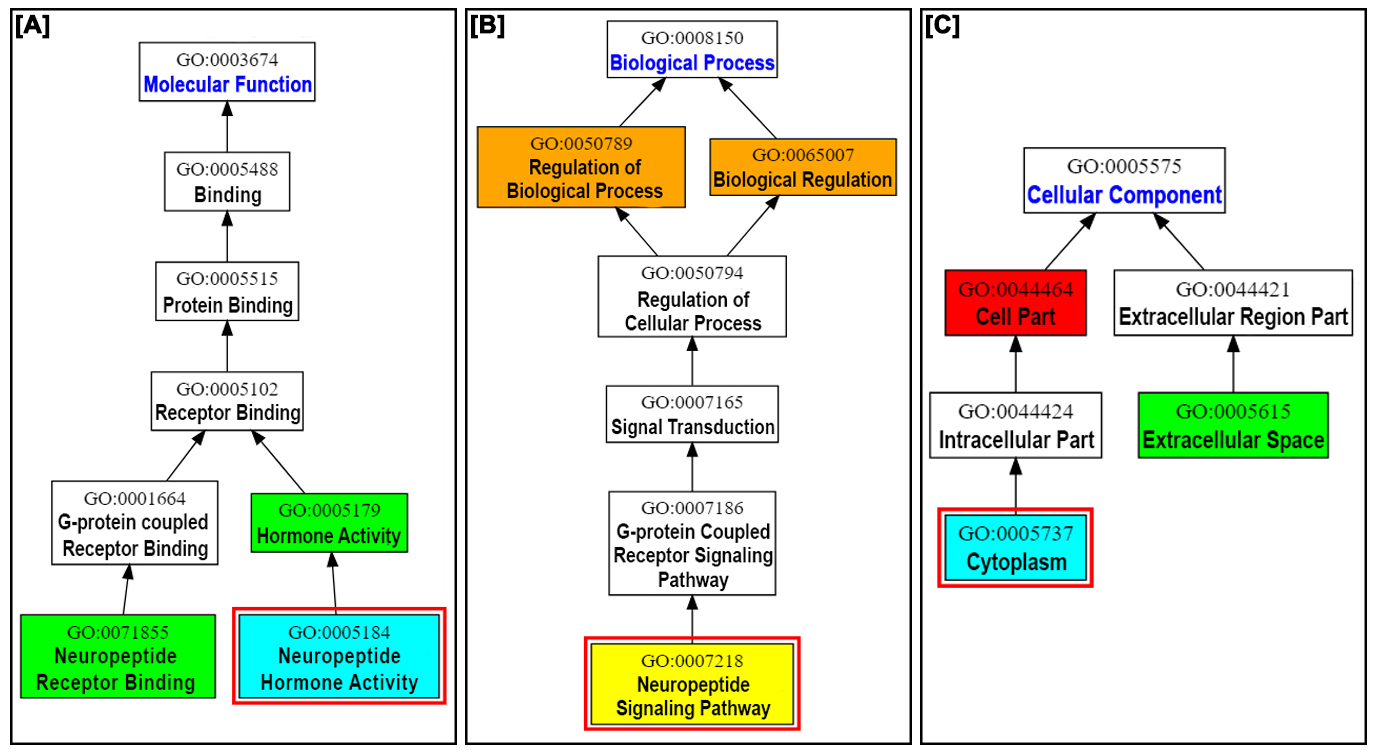

Supplement: Supplementary file 1 [file biomolecules-13-00109-s001.zip › Supplementary Figure S1.jpg]

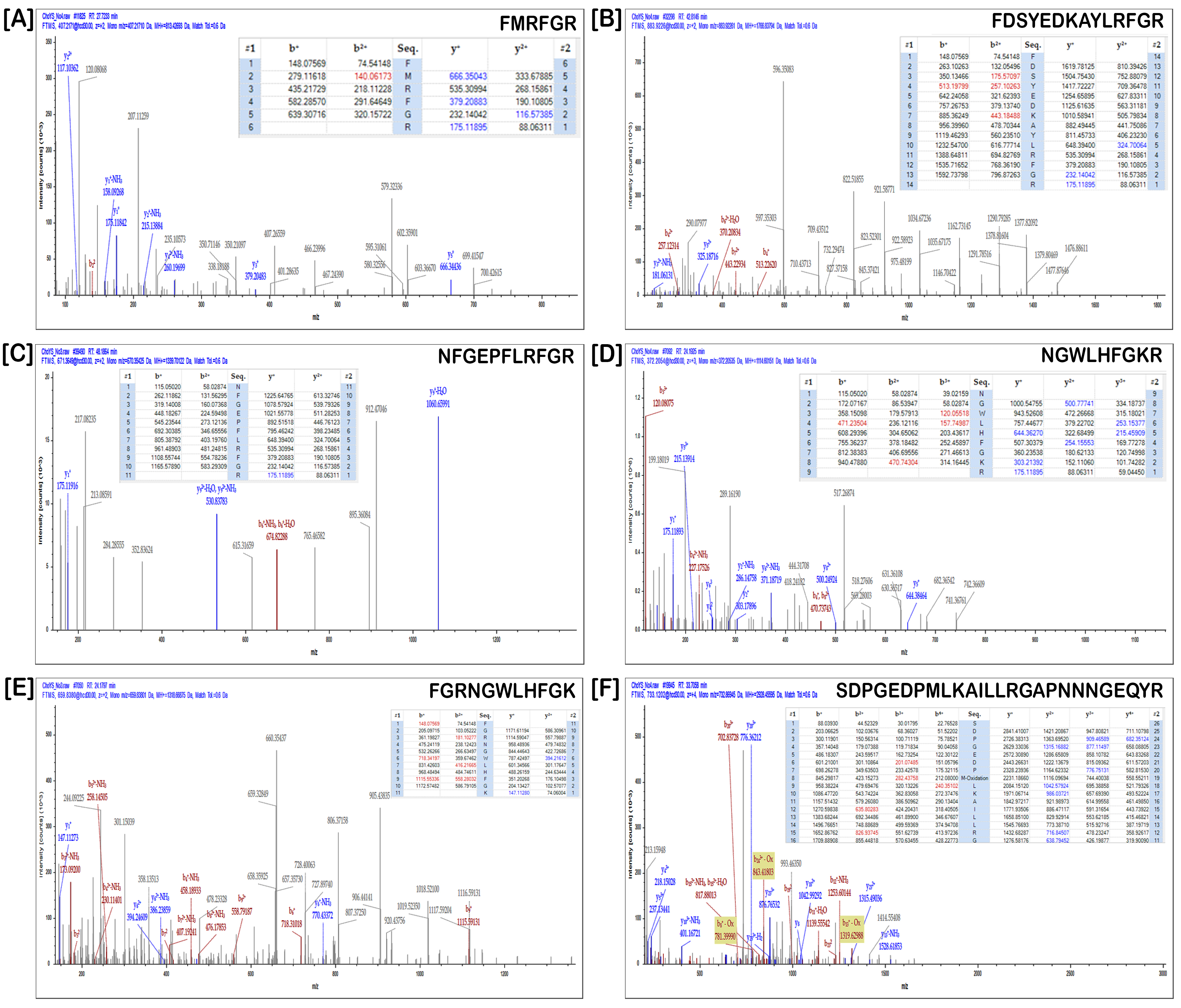

Supplement: Supplementary file 1 [file biomolecules-13-00109-s001.zip › Supplementary Figure S2.jpg]
